# Abstracts and Gleanings

**Published:** 1888-12

**Authors:** 


### ABSTRACTS AND GLEANINGS.

**Shock.**—David W. Cheever, M. D., Boston *Medical Journal*, writes: Moderate shock terminates in reaction This is the *recoil* of the system. It restores the balance; but the pendulum which marks the nervous force swings back beyond the normal line. We have temporary fever, flush, full pulse, excitement.

Severe shock is more lasting. The pulse vibrates, intermits, flags, rallies, flags again, is soft, compressible, uncertain; faintness is constant, but partial; vomiting occurs; cold extremities; dilatable pupils; pallor; imperfect reaction; very slow recovery; a condition where a feather turns the scale against the patient.

If now an operation is done, we have renewed shock, prolonged shock, secondary shock; a matter of days rather than hours; persistent nausea; exhaustion; a lowered temperature; diarrhœa; imperceptible and gent e death. Or, if an old person, that state known as prostration with excitement; typhoid delirium, a dusky flush over the malar bones, dull eyes, intermittent pulse, jactitation, exhaustion, death.

Primary shock; prostration, nausea, excitement, collapse. Loss of blood, from accident or operation, adds to the shock or complicates its symptoms.

Jar, crushing, mutilation, pain, cutting, bleeding, chilling, all act on the nervous center; react on the gangalia, the heart, the power of breathing, the temperature, the consciousness, the life.

Given then the problem and the phenomena of shock, what particular influences have the operative procedures of *modern* surgery upon them?

They may be summed up in three points: The effects of anæsthetics; the effects of the operations; the effects of the dressings.

These all belong together and effect each other.

Anæsthetics annul pain, but end in nausea.

Operations under anæsthetics are needlessly prolonged and exhausting.

Modern dressings are tedious and chilling.

Have we lessened, or added, shock by modern surgery?

Pain and bleeding are *less*. Slow cutting, nausea, exposure, low temperature are *more*. Primary shock is diminished; secondary shock is increased.

Formerly the time consumed in an operation was short. An amputation was hurried, now it is deliberate; an abscess was incised, now it is aspirated and curetted; a joint injury was cut off, now it is excised; the peritoneum was peeped into, now it is boldly explored; the bladder was cut off for stone, now it is a prolonged crushing and washing; a breast was amputated, now the axilla is formally dissected. The old method was a matter of minutes; now it is one of hours.

Patients are frequently from one and a half to two hours on the operating-table; and three hours in recovering consciousness so that they can swallow. Do we realize what this prolonged cutting, pinching, and dissecting mean to the nervous system after anæsthesia is past? Does not the long exposure of the great veins to the air, in dissecting tumors, increase coagulability and future infraction? Can the peripheral nerves be lacerated *seriatim* without exhausting their constitutional irritability? It is recognized that long-continued and large dissections on the front and sides of the neck are especially fatal.

Operations of *secondary* magnitude are now so prolonged that I have repeatedly seen patients die of primary shock, or *repeated shocks*, where the patient was one to two hours under the knife. It is said that he had not the vitality to resist. He had not; but consider what a perineal section, scooping out a uterine tumor, curetting a bladder, removing glandular enlargements, sometimes involve in time, in exhaustion, in capillary oozing, in shock.

Equally unphilosophical and fatal is the practice of operating in cases of primary shock before reaction has come on. An amputation is begun in half-life, and ended in death. Especially difficult to decide are the cases where the patient reacts imperfectly and relapses. These cases are easily made fatal, and only saved by quick amputation, slight exposure and short anæsthesia. The golden moment of fairly established reaction must be seized, before traumatie fever sets in This moment comes in from six to eighteen hours after the injury, or it never comes.

It should be considered on *axiom* that anæsthesia does not diminish existing shock, but only annuls the additional shock which the pain of cutting produces. It prevents the pain of an operation from increasing the shock which may be present from injury. It prevents the pathological case from experiencing the shock produced by the pain of cutting out a tumor; it does *not* prevent the secondary shock of the multilation; it adds to secondary shock if the anæsthesia is prolonged.—*American Practitioner and News*.

**The Turkish Bath.**—Most of those who indulge from time to time in the luxury of a Turkish bath have need to observe a discreet moderation in its use if they would reap its advantages without incurring its occasional risks For the majority of persons it is, with this proviso, a wholesome and enjoyable aid to cleanliness. The rheumatic, the gouty, and the dyspeptic have often proved its therapeutic value, and the reason for this is not far to seek. Its superior efficiency as a cleanser of the skin surface, and its powerful diaphoretic action, need only be mentioned in order to commend its use in such cases. The action set up is not, indeed, a purely local one. Not only is the skin excretion vigorously stimulated, but the blood vessels, the absorbents, and the deeper tissues generally, are washed by the current of outgoing fluid thus set in motion, while at the same time the strain on the frequently overburdened kidneys is lightened, and this organ rendered proportionately freer and fitter for the discharge of its duties. We must not allow ourselves, however, to view the matter in its most favorable aspect alone. Serious mishaps do occasionally occur in the Turkish bath, and of this fact the recent sudden death of a man in one of the northern counties affords a suggestive illustration. The deceased had been a heavy drinker, and suffered from fatty degeneration of the heart. He had been advised by a medical man that he should not enter the bath, but in spite of the warning did so, and appears to have spent the greater part of the night in the hot and cold rooms alternately. It should be mentioned that about the same time he indulged, though not very freely, in drining whisky. A few hours later he was found dead in the cooling-room. We may remark, in passing, the obvious need of limitation in the time during which the establishment is allowed to remain open. In this case the immediate cause of death was clear enough to show that the danger in such cases, and it is a real one, depends on the bather's health at the time. It is generally allowed that weakness of the heart muscle from actual disease contra-indicates a bath of this kind. There are also cases, however, in which almost equal caution is necessary, though the cause of weakness is mere exhaustion from temporary overstrain. This is a point to which the worried, the anxious, and the overworked would do well to give a due share of attention.—*Lancet.*

**The German Treatment of Obesity.**—Reference is had to the Schweninger, or rather the Oertel method. The following are the objects aimed at in this cure:

1. To improve the muscular tone of the heart.
2. To maintain the normal composit'on of the blood.
3. To regulate the quantity of fluid in the body.
4. To prevent the deposit of fat.

These objects are obtained by the following means:

1. The muscle of the heart is strengthened by enforced exercise, such as climbing heights. This requires great care, and the exercises must be graded, the amount of work being increased as the patient can bear it.

To preserve the normal composition of the blood, the food should be chiefly albuminous. It may consist of the lean of roast, or boiled beef, veal, mutton, game and eggs. Green vegetables (as cabbage or spinach) may be taken; fat and carbo-hydrates only in very limited quantities; from four to six ounces of bread per diem.

3. To regulate the qantity of fluid in the body the amount of fluid drunk daily must be limited. One cup (rather less than six ounces) of coffee, tea or milk morning and evening, and about twelve ounces of wine, and from eight to sixteen ounces of water shall comprise all the fluid consumed in twenty-four hours. Beer is entirely forbidden. The discharge of the fluid from the body is promoted by active exercise and occasionally by a course of baths, with packing.

4 To prevent the deposit of fat the principles of diet already set forth must be carried into practice as follows:

*Morning.*—One cup of tea or coffee with a little milk, altogether about six ounces; bread, about three ounces.

*Noon.*—Three to four ounces of soup, seven to eight ounces of roast or boiled beef, veal, game, salad or a lighter vegetable, a little fish (cooked without fat) if desired, one ounce of bread or farinaceous pudding (never more than three ounces), three to six ounces of fruit, fresh preferred, for desert. It is desirable at this meal to avoid taking fluids, but in hot weather, or in the absence of fruits, six to eight ounces of light wine may be taken.

*Afternoon.*—The same amount of coffee or tea as in the morning, with, at most, six ounces of water, an ounce of bread as an exceptional indulgence.

*Evening.*—One or two soft-boiled eggs, an ounce of bread, perhaps, a small slice of cheese. Salad and fruit, six to eight ounces of wine, with four to five ounces of water.—*Epitome.*

**Forty-three Gall Stones Passed by a Child two and a half Years Old—Recovery.**—Dr. J. K. Cantrell, Alton, Mo: I was called on March 9 to see a child of Mr. Smith Warns, aged two and a half years. The mother informed me that the child had been suffering from worms all winter, and she had been giving it oil and turpentine, which seemed to relieve it for some little time. I made a slight examination, and arrived at the conclusion that the mother's opinion was correct. I left the following prescription:

℞. Hydr. chl. mit.............................gr. xii,

Santonine.................................gr. ij.

M. ft. chts. No. 4. One powder to be taken every three hours, followed by oil and turpentine.

On the eleventh, the father call d at my office and informed me of the child's improvement, but that no worms were expelled. When I visited the child I thought I discerned evidences of jaundice, but she presented no alarming symptoms. On March 21 I was hastily summoned, to find her in convulsions, which I soon relieved by a few doses of chloral hydrate and potassium bromide. I also left the same prescription as before, somewhat increasing the dose of santonine. At the same time I learned that food did not agree with it, and that the urine had been scant for three or four weeks, saffron-colored, leaving its impress upon the clothing, the skin and conjunctiva. To relieve these conditions I prescribed:

℞. Soda sulphite..............................ʒ ij,

Aquæ.......................................℥ iij.

M. S.—Teaspoonful every three or four hours.

Also:

℞. Potass. bicarb.............................ʒ ss,

Aquæ.......................................℥ iij.

M. S.—Tablespoonful every four hours.

I was led to believe there was an obstruction in the gall-duct, and by palpation over the liver and cystic duct, the child complained of pain over the liver, extending to the stomach. The mercury and santonine were given with a view of depleting the portal circulation, and expelling ascarides if any were present. I

requested the father to call upon me whenever he thought it necessary. On the 29th of the same month the father brought me a tin can containing twenty-seven gall-stones, some of which were as large as a pea, others as large as an ordinary marble. They had been discharged with the stools every day since the powders had acted. Some smaller ones could not be preserved. The last ones discharged were covered with thick inspissated mucus, others almost dry. I dissolved one, and it was mixed with a gray and greasy substance. The father was requested to keep a lookout for more. The child cried at each stool, almost culminating in convulsions.

On June 13 the father returned, requesting more medicine, as the child was suffering as before. Feeling that my former prescription had been a success, I repeated the mercury and santonine, to be followed by oil and turpentine. On June 17 I received nineteen more, but none of them as large as the largest of the previous ones. Some of them had softened, and were almost flat in shape. The child's urine was very dark and scanty, for which I prescribed 10-grain doses of potass. bicarb. in a half glass of water, three or four times a day. In addition, I ordered:

| | | |
|---|---|---|
| R. | Mag. sulph. | ℥ j, |
| | Mag. carb | gr. vij, |
| | Spir. ammon., arom | gtts. xv, |
| | Aq. pura | ℥ x. |
| M. | S.—Half to be taken morning and night. | |

The above was given for six days, and at the end of that time I received seven calculi, three of which were as large as the largest of the first passed. The child had made no complaint when these were passed. That she was looking much better, the skin was clear, her food agreed with her better, and the urine was clear and ample. On August 4, I was informed of the full recovery of my patient.

This case may serve as a guide to others, when surprised at a hitherto accepted improbability.—*Epitome.*

**Hot Air Inhalations for Phthisis.**—The therapeutics of phthisis which may be based on the bacillary theory of the causation of the disease, must consist in the employment of means calculated to destroy the germs in their habitat. The difficulty which has been thus far encountered is in the fact that the agent which will destroy the germ will also destroy the integrity of the tissue, and even the life of the individual. The agent which will enter the system and destroy within it, without injury to the individual, the cause of the disease is the desideratum which is anxiously awaited. The *Lancet* refers to the investigations of two German observers, or, to speak more correctly, two observers in Germany, who have, independently of one another, been engaged in investigations on the bactericidal property, of heated dry air, and on the methods of utilizing this property for the practical treatment of phthisical patients. Dr. Weigert, who appears to be an American living in Berlin, finding that the tubercle bacilli outside the body die at a temperature of 41° C. (106° F.), and are adversely affected by one of 38° (100.4° F.), had constructed all apparatus for the inhalation of heated air, and commenced to make trials on phthisical patients in the early stage, recommended to him by other medical men, he himself not being in practice. At first a temperature of from 40° to 60° C. (104° to 140° F.) was employed, the air for inhalation being quite dry. This temperature was gradually raised as high as 80° C. (176° F.) The patients bore this hot air exceedingly well, and continued to inhale it for three or four hours a day during a month, the only unpleasant effects produced being hyperæmia and dryness of the mucous membrane. The general effects are represented as having been remarkable, patients who had been falling away picking up strength and becoming quite robust, the physical examination showing at the same time that the dullness and rales had perceptibly decreased. The bacilli in the sputum, which had been very numerous, rapidly diminished in number, and finally disappeared altogether. These observations were confirmed by several other medical men. Dr. Halter, of Lengerich, Westphalia, seems to have gone even further than Dr. Weigert, he having himself inhaled, and caused patients also to inhale, dry air heated to 90° C. (194° F.), with satisfactory results.—*Medical Age.*

**Monthly Summary of Medical Progress.**—Dr. W. S. Wells, in *Popular Science News:* At the recent meeting of the French Association for the "Advancement of Science" (*l' Union Med.*) M. Gillot communicated an article regarding the tongue as a guide to the diagnosis of lesions of the intra-cranial blood-vessels.

In the ordinary examinations of the tongue only the upper surface is seldom noticed.

Dr. Gillot refers to this neglect, and asserts that the under surface may present certain points of diagnostic significance.

In a young and healthy person the veins alone are prominent beneath the mucous membrane; but with the advance of age, or as a result of disease, these veins become dilated, tortuous, or varicose, and the venules and capillaries become visible. In many cases little dilatations, like grains of sand, may be seen on the smaller vessels, and may be few and disseminated, or numerous and grouped together. They are usually situated a short distance from the tip of the tongue, on either side of the medium line, or near the root of the organ.

Their color varies, according to their size and the condition of the general circulation, from a bright red to purple or almost black.

These projections, as claimed by Dr. Gillot, are true miliary aneurisms, caused by a thinning of the walls of the vessels, and are analogous to the aneurisms occurring on the blood-vessels of the brain. He believes that the existence of these dilatations of the blood-vessels of the under surface of the tongue is diagnostic of a similar condition of the blood-vessels of the brain. The circulation of the tongue has very close relations within the cranium—the same influences which act upon the one, acting also upon the other—and the inspection of the under surface of the tongue furnishes as valuable an indication of the state of the cerebral circulation as does the examination of the cavity of the eye by the ophthalmoscope.

Dr. Gillot refers the primary cause of these miliary aneurisms of both the tongue and the brain to the so-called arthritic diathesis, and says he has never seen these dilatations in any but those suffering from gout, rheumatism, gravel and heart diseases of arthritic origin. He therefore urges in the aged, and those presenting rheumatic and gouty symptoms, a careful inspection of the under surface of the tongue, as likely to assist materially in averting, by proper treatment, grave cerebral disorders.—*P. S. News.*

**M Schwartz** lately read to the Surgical Society of Paris a communication on a case of pressure paralysis from a callus, reported by M. Marchand. A man aged twenty-nine had been thrown from a horse, sustaining a fracture of the upper end of the fibula, with posterior luxation of the tibia. A plaster-of-Paris splint was applied; but when it was removed, at the end of six weeks, the patient was found to be suffering from various disturbances of sensibility and motion.

The foot was in an equinos position, and anæsthesia and paralysis existed in the parts supplied by the external popliteal nerve. The electrical contractility was abolished, and a marked degree of atrophy existed.

There was considerable callus at the seat of the fracture of the fibula, and pressure here caused pain. Thinking that the nerve might be imprisoned in the callus, M. Marchand laid bare the tumor, and discovered the external popliteal nerve passing over it. On following up the trunk of the nerve, it was found to be embeded in a mass of hard fibroid tissue.

When freed from this, the nerve was found enlarged at this part, and congested.

The wound healed readily, but it was only gradually that motion and sensation returned; and it was remarked as singular that the power of voluntary movement was gained before the restoration of electrical contractility. At the end of seven months the patient's condition was normal.

M. Marchand referred to the rarity of this accident, but called attention especially to the exceptionally good results following the release of the imprisoned nerve. In some of the cases previously reported, slight or no improvement was obtained by operation, and in others inflammation of the nerve occurred, necessitating amputation.

**Another** case is published (*International Journal Surg.*) of preservation of several digits, this one being remarkable for the length of time which elapsed from the time of the accident until the adjustment of the part.

A boy while splitting wood cut off a part of his right thumb. It dropped to the ground, and he ran into the house. The mother hunted up the father, who went to the barn, harnessed the horse, found the severed piece of thumb, wrapped it with the stump in a cloth, and drove nearly three miles to the doctor. Fully an hour and a half elapsed before the doctor saw him, when he removed the cloth, and found the thumb lying loosely in the bandage, and covered with sand and dirt. The doctor cleansed it and secured it to the stump. It united promptly, and two years afterwards it was hard to tell which thumb had been injured.—*Pop. S. News.*

**Dr. W. J. Clapp** (*London Lancet*) reports a case of tetanus successfully treated with strophanthus. The patient was a man aged twenty-three. Three weeks previously he had an accident by which he suffered severe pain; followed, in time, by burning pains between the shoulders, extending down the spine Subsequently followed rigidity of the abdominal muscles, spasms of body, chest, arms, thighs, and legs. The jaws became locked, countenance anxious, face and mouth contracted, pulse quick and wiry, temperature 108° F. The urine was dark and scanty, without deposit; bowels constipated.

Various remedies were with difficulty given, without improvement; and it was determined to give strophanthus a trial. Dr. Clapp employed tabloids containing 2 minims each. One was given every three hours, it being with difficulty placed in his mouth, and cold water, also, after each tabloid. The second day after this treatment with strophanthus a decided improvement was noted. The doctor could open the patient's mouth sufficiently to introduce the mouth of a feeding-cup. The spasms in all the affected parts became less frequent, the pulse quiet, and the temperature lower. Nourishment was increased. The frequency of the doses of strophanthus was gradually lowered. The urine became copious and clear, all the symptoms abated, and in a fortnight the man was able to walk and take food as usual.—*P. S. News.*

**Burns** being so common an accident, and often so difficult to treat, the following formula is recommended by an exchange:

| | | |
|---|---|---|
| ℞. Tannin | grams | iv, |
| Alcohol (95°) | " | iv, |
| Ether sulph. rect | " | xxx. |

M. Paint the parts with this two or three times a day.

After evaporation of the ether, there remains a fine pellicle of tannin over the burn, which excludes the air, and takes away the pain and inflammation, and the cure is much more rapid than with the various collodion preparations. The first painting of the parts should always be preceded by a careful antiseptic washing, to remove foreign substances that may have adhered, and all blisters should be punctured before applying the remedy.

If sometime has passed before treatment, a slight coating of iodoform should be powdered over the part first.

**Antipyrin in Pertussis.**—Antipyrin seems to be the most satisfactory of the very many suggested remedies for whooping cough. Reports are multiplying as to its usefulness. Though not a specific, it seems in the majority of cases to exert a beneficial action, shortening the duration of the disease and mitigating the severity of the paroxysms. A practitioner has now made use of this remedy in six cases with the most excellent results. In an infant of one year, given at the beginning of the second stage, doses of gr. ⅓, four times daily, its effect seemed magical to the parents. The paroxysmal element entirely disappeared by the fourth day and a speedy recovery took place. The results in the other cases, though less rapid, were satisfactory.—*Polyclinic.*

**Corrosive Sublimate as a Practical Disinfectant.**—Our knowledge regarding the relative value of disinfectants as measured by their power to destroy micro-organisms dates from the researches of Robert Koch, published in 1881. Koch found that most micro organisms are destroyed by a solution of corrosive sublimate of the strength of 1:5000; while a solution of the strength o 1:1000 is fatal to all. He also found that corrosive sublimate has a decided superiority over all other substances as an antiseptic. A solution of the strength 1:1000000 had a marked restraining power on the germination of the spores of the *B. anthracis*, for example, while a solution of the strength of 1:3000000 prevented their development. The results of other investigators, while showing that antiseptic and disinfecting powers are possibly not quite so great as claimed for it by Koch, still confirm the latter so far as to show that they are considerably greater than those of any other known substance, with the possible exception of one or two other salts of mercury, which for one reason or another are not so available.

As a result of these investigations, and more directly following the recommendations of the Committee on Disinfectants of the American Public Health Association, published in 1885, corrosive sublimate has taken the place, to a large extent, of all other disinfectants, for nearly every purpose excepting aerial disinfection. Every State board of health to whose reports, for the past four or five years, I have been able to obtain access, has published the recommendations of this committee, thus giving them a wide circulation, and nearly every one has distinctly indorsed them as representing the best methods of disinfection known to us. The Board of Health of Maine alone objects to corrosive sublimate, on account of its poisonous properties, and because it forms an insoluble compound with albumen. Figures showing the exact extent to which it is used, can be obtained, if at all; only through local boards of health, and the reports of these are not easily accessible. There were used, however, in the city of Boston, according to the reports of the board of health, in 1884, 850 pounds; in 1885, 1550 pounds; in 1886, 1400 pounds, and in 1887, 2250 pounds of corrosive sublimate for purposes of disinfection.—*Sanitarian.*

**Lipanine**, a substitute for cod liver oil, is a substance made by Kahlbahm, of Berlin, by partial saponification of olive oil. Kohts claims that he has been pleased with the results of its use in his children's clinic in Strassburg.—*Pac. Rec.*

**Headache.**—The following table of the different kinds of headache and the treatment is given by Dr. Benj. Edson in *Medical World:*

| FORM. | TREATMENT SUGGESTED. |
|---|---|
| 1. Anemic. | Amyl., bella., f.; menthol, quin. |
| 2. Bilious. | Ammon. chl., hg., mag. sulph., podoph. |
| 3. Cardiac. | Cactus, dig., glonoin., strophanth. |
| 4. Catarrhal. | Acon., potas. iod , puls., duboisine. |
| 5. Cerebral softening. | Anodyne, ergot. |
| 6. Cerebral tumor. | Ergot, potass. iod. |
| 7. Cinchonism. | Bromides, iodides. |
| 8. Congestive, active. | Acon., erg , morph., pop., salines. |
| 9. Congestive, passive. | Aq. ferv., dig., potass. acetat. |
| 10. Climateric. | Cimicif., ver. vir., sulphur. |
| 11. Constipation. | Aloin, cascara, hg , nux vom , pod. |
| 12. Diabetic. | Potass. brom., valerian. |
| 13. Dyspeptic. | Byron, guarana, nux vom., sod., salicyl. |
| 14. Fevers. | Acon , antipy., dig. ge'., ver. vir. |
| 15. Gouty. | Colch., salicyl. |
| 16. Hemicrania. | Antipy., as., can. ind., dig., gels. |
| 17. Hemorrhoidal. | Cascara, sulphur. |
| 18. Hysterial. | Asaf., camph., hyosc., val of zn. |
| 19. Idiopathic. | Caffeine, guarana, bromides. |
| 20. Inebriate. | B omides, camph. monobrom., chloral, hyosc., pilocarp. |
| 21. Migraine. | Antipy., brom., ergot, glonoin, menthol. |
| 22. Meningeal. | As. et op., frlgus, gels. |
| 23. Menstrual. | Am. mur., antipy., gels., picrot., virburn. |
| 24. Malarial. | Ars. et. bell., gels., quin., am. picr. |
| 25. Nervous. | Bromides, gels., guarana, strych., zinc. |
| 26. Neuralgic. | Antipy., caffein, gels., phosp., quin. |
| 27. Optical. | Correct the vision. Rest. |
| 28. Ovarian. | Am. mur., gels., vibufrn. |
| 29. Periosteal. | Potass., iod. |
| 30. Periodical. | Ars , can. ind., gels., quin. |
| 31. Plethoric. | Alkalies, laxatives. |
| 32. Rheumatic. | Colch., pot. ind., salicyl. |
| 33. Sick. | Caff citrat., guarana, nux vom., sod., phosph. |
| 34. Syphilitic. | Hg., pot., iod., stillingia. |
| 35. Toxemic. | *p. r. n.* |
| 36. Uræmic. | Elater, morph., pilocarp., podoph. |
| 37. Uterine. | Bella., cimicif., viburn. |
| 38. Worms. | Salicyl., vermicides, vermifuges. |
| 39. Zymotic. | *p. r. n.* |

**Catarrh Cures.**—A writer in the *Weekly Medical Review* says: "I have collected every catarrh, asthma, and hay-fever 'sure cure' that is in the market, numbering in all fifty-eight, and have carefully examined them. Eighteen of these 'sure cures' are bold-faced frauds One ounce of quassia chips, a pound of table salt, forty gallons of water, will make one barrel of 'sure cure,' that sells for one dollar a bottle, holding six ounces. The same quantity of water, a pound of muriate of ammonia, a pound of ground cubebs, and a little common potash will make another 'cure' that sells for fifty cents a bottle, holding four ounces. These two are the best of the eighteen frauds."—*Sanitarian*.

**The Venom of the Rattlesnake and the Gila Monster.**—In some recent numbers of that very lively publication known as *Forest and Stream* there has been a series of very clever (*Anglice*) articles by Dr. Dr. H. C. Yarrow upon the poisonous reptiles of America, especially the rattlesnake and the Gila monster, *Heloderma suspectum*. In the chapters devoted to the discussion of the rattlesnake we find nothing that seem to require mention here except in regard to the treatment. It will be remembered that De Lacerda some years ago asserted that the permanganate of potassium is an antidote to rattlesnake venom, and that Mitchell and Reichert found that when the permanganate is mixed with the venom in a beaker glass it destroys its constitution. The value of permanganate as a practical antidote in snake-bite was studied by Dr. Yarrow by injecting the venom into the body of the animal, and shortly afterwards injecting permanganate of potassium in immediate proximity to the original wound. A very important observation was made that if a litgature be placed around the leg of an animal, and a certain quantity of venom be injected below the ligature, followed by a solution of the permanganate, no poisoning resulted, but even a five per cent. solution of the permanganate was of no value if there were no ligature above the point of injection, although the permanganate injection followed the venom in less than half a minute. The number of experiments reported by Dr. Yarrow does not seem to us sufficient to allow of a positive conclusion, but they indicate that the permanganate is useful in snake bite when the bite is situated where it can be isolated by a ligature, and his directions "tie a ligature tightly around the wounded part, incise it freely, and then inject as soon as possible permanganate of potassium into the tissues round the wound," is justifiable.—*Therapeutic Gazette*.

**Collinsonia.**—We desire to again call the attention of our readers to the use of this most valuable agent in the treatment of conditions in which the veins are involved. In 15-drop doses, four or five times daily, it will prove invaluable and superior to any known remedy in the treatment of varicosed veins, wherever found and from whatever cause. It is of value in phlegmasia dolens with other appropriate remedies. In the treatment of hemorrhoids it certainly has no equal. It is a valuable tonic in enfeebled heart with a general relaxed condition of the circulatory system. It will relieve incontinence of the urine in enfeebled and poorly nourished children. It is of value in gonorrhœa, in many cases acting most speedily. As a stomach tonic, it can be relied upon, and when combined with hydrastis it has no superior. It will relieve pain quickly in many cases of gastralgia.—*Chicago Medical Times*.

**Report of the Delegate to the Congress for the Study of Tuberculosis.**—Dr R. C. M. Page, at *N. Y. Academy of Medicine*, made the report which had received the verbal approval of Villemin and Landouzy. They had found that true tubercle generally contains the bacillus, which was first supposed to exist by Bouchard, and afterwards discovered by Koch. All other substances occurring in non-tuberculous subjects do not contain this bacillus. T e bacillus remains virulent in running water at a temperature of 60° F. for six weeks, and in dried sputa for months. It is the cause, not the result of tuberculosis. The disease is contagious under certain conditions, especially in conditions that tend to produce lower vitality. It is transmitted through cow's milk, beef, etc. In the diagnosis of pulmonary tuberculosis in doubtful cases, the bacillus should be sought for; but it may not be found in the early stage before it has become liberated. As a further test, a lower animal may be inoculated with some of the patient's sputa, and the bacillus be sought for subsequently. No practical means for destroying the bacillus in patients has yet been discovered. Treatment at present is prophylactic—by attention to climate, food and exercise, and by medicines directed against symptoms as they arise.

The report of the delegate to the British Medical Association was also read.

**The Treatment of Ulcers.**—An article appeared in the *London Medical Record* (December 15, 1887) giving interesting details of ulcers by phosphoric acid, as shown by the experience of Dr. Grossich. By this method of treatment, he used a 10 per cent. solution of pure phosphoric acid in distilled water. The ulcer is covered with a bit of lint dipped in this solution, and the dressing renewed three or four times a day. The patient, for the first few minutes, feels a slight burning sensation, but this soon passes, and within twenty-four or thirty-six hours the ulcer cleans, and looks better. Inflammation or eczema of the surrounding parts disappears, and all pruritus ceases. The ulcer cicatrizes rapidly, and the cicatrix is firm and healthy.

Kollischer treated tubercular affections of the joints with injections of the phosphate of lime, with great success. Dr. Grossich has also had good results with this treatment, and cites some very interesting succesful cases.

The treatment by the solution of phosphoric acid was further employed in a case of tuerculous abscess of eight months' duration and also a case of eczema marginatum which had lasted more than a year, and good results followed.

**The Purgative Action of Glycerin Enemata.**—Dr. Subbotic confirms the testimony which has rapidly accumulated as to the efficaciousness of a rectal injection of glycerin in producing evacuation of the bowels. He offers an explanation, however, of the occasional failure which follows its employment from the fact that it produces no result when injected into an entirely empty rectum. When, however, masses of fæces occupy the rectum the glycerin injection is invariably promptly active. The injection acts through an increased secretion of fluid and a consequent irritation of the intestine. This irritation, however, appears only to be sufficient to empty the lower parts of the intestine, while it is insufficient when the rectum is empty to cause the downward passage of the contents of the upper bowels.—*Centralblatt fur Gynakalogie.*

**Suture of a Wound of the Liver.**—The *Riforma Medica* (April 25) states that Prof. Postempski recently stitched up an incised wound of the liver. The operation, which is said to be the first of the kind ever performed, took p'ace in the Ospedale della Consolazine at Rome, on April 8th. The abdomen was opened and the edge ot the wound, which was situated in the left lobe, and which was two and three quarter inches in length and one-third inch in depth, were brought together by catgut sutures, applied by means of extremely fine needles. The hemorrhage, which had been very free, was ot once checked, when the wound in the liver-subtance was closed. On April 23, four days after the operation, the patient's temperature was normal and he was doing well. Prof. Postempski will publish the case in detail in due course.—*Med. Register.*

**Iodoform and Tar.**—Dr. S. Ehrmann combines tar and iodoform in such a way that the particles only show hyaline plates under the lens, the crystalline plates of iodoform not being recognizable. This "bituminate of iodoform" resembles mica; the translucent scales of the mixture are easily pulverized. The odor of iodoform is wholly absent; only a mildly aromatic and not unpleasant smell of tar is perceived, and a trace of liquid storax destroys this. It is used with success in the treatment of soft sores as a dressing, slso after opening supurating buboes, and in gummy tumors and ulcers of the leg Only profuse secretions will develop the odor of iodoform.—*Ind. Med. Jour.*

**In Alabama**, a black negro girl about eighteen years old, has given birth to twins at seven months, one of which is as "black as the ace of spades," and the other as white as any white child her medical attendant ever saw. This is as puzzling as the case recently reported, in which a beautiful young woman with a tinge of negro blood so slight as to be imperceptible, married an unsuspecting white gentleman, and in due time presented him with a black baby.

**Canadol as an Anæsthetic.**—The substance named is a hydrocarbon distilled from American naphtha. It is transparent, volatile, insoluble in water and alcohol, and has a benzine like odour. When applied to the surface of the body it produces a limited reduction of temperature, and an anæsthesia which is said to be complete in sixty seconds. It is recommended for the painless performance of minor operations by Dr. Plionchkine in the *Rev. d. Therap. Med.-Chirurg.*

